# Therapeutic efficacy of fecal microbiota transplantation in severe food intolerance: a case report

**DOI:** 10.3389/fnut.2025.1594022

**Published:** 2025-05-19

**Authors:** Yanhui Huang, Jiayuan Huang, Yuange Li, Tianyu Xu, Guoqiao Quan, Peihao Xu, Xiaoya Yang, Zhou Liu, Wenrui Xie

**Affiliations:** ^1^Department of Gastroenterology, Research Center for Engineering Techniques of Microbiota-Targeted Therapies of Guangdong, The First Affiliated Hospital of Guangdong Pharmaceutical University, Guangzhou, China; ^2^School of Clinical Medicine, Guangdong Medical University, Zhanjiang, China; ^3^Department of Physiology, Guangzhou Health Science College, Guangzhou, China; ^4^Department of Neurology, Affiliated Hospital of Guangdong Medical University, Zhanjiang, Guangdong, China

**Keywords:** food intolerance, fecal microbiota transplantation, gut microbiota, autism spectrum disorder, gastrointestinal symptoms

## Abstract

This report presents the first documented application of fecal microbiota transplantation (FMT) for the management of extensive multi-food intolerance involving 52 specific foods in a pediatric patient with autism spectrum disorder (ASD). A 7 years-old autistic child was diagnosed with food intolerance to 52 items, presenting with generalized rashes, diarrhea, and malnutrition (BMI of 12.9) upon exposure or ingestion of the implicated foods. The child received oral fecal microbiota capsule treatment, with a daily dose of nine capsules (a total of 120 capsules per course) for two consecutive treatment courses. The rashes resolved, the child regained tolerance to previously intolerable foods, nutritional status improved, and stool consistency normalized. This case suggests that FMT may hold therapeutic potential for managing food intolerance in autistic patients.

## 1 Introduction

Food intolerance (FI) refers to non-immune-mediated adverse reactions to food or food components at normal tolerated doses, including metabolic, toxic, pharmacological, and undefined mechanisms (1–6). It’s prevalence ranges from 15% to 45% (7). The main mechanisms underlying FI include these following aspects: Firstly, Certain foods contain pharmacologically active com-pounds (e.g., histamine, monosodium glutamate, caffeine) that can induce physiological responses like smooth muscle contraction and inflammation, contributing to FI (8). Secondly, Lactase deficiency is a major cause of lactose. Similarly, deficiencies in histamine-degrading enzymes, such as diamine oxidase (DAO) and histamine-N-methyltransferase (HNMT), can lead to histamine accumulation and FI symptoms (9). What’s more, Food additives and preservatives (e.g., nitrates, nitrites) may also induce FI reactions (10, 11). The precise mechanisms remain unclear, but these components can trigger adverse reactions in susceptible individuals. Diagnosis of FI requires detailed medical history, dietary and lifestyle assessments, and laboratory tests (e.g., blood and stool analyses) or imaging to exclude organic diseases and food allergies (FA) (12). Currently, the double-blind, placebo-controlled oral challenge (DBPCFC) is considered the gold standard for FI diagnosis, involving food elimination and gradual reintroduction to identify triggers (13). Food-specific IgG or IgG4 serological testing is widely used in clinical practice (14).

The gut microbiota plays a crucial role in the pathogenesis of FI. FI is often associated with gut dysbiosis, characterized by the reduction or increase of specific microbial populations, leading to impaired gut barrier function, enhanced inflammatory responses (5, 15), and abnormal metabolic functions. FMT, as a therapeutic approach to modulate the gut microbiota, may restore microbial balance and improve FI symptoms by transplanting healthy donor microbiota into the recipient. Annabel Clancy and Thomas Borodyhave (16) demonstrated that FMT can significantly alleviate FI symptoms in patients with irritable bowel syndrome (IBS), highlighting the potential therapeutic value of gut microbiota in FI. FMT can also improve both autism symptoms and gastrointestinal symptoms in patients with ASD (17).

To date, there have been no reports on improvements in food intolerances in patients with ASD treated with FMT. Therefore, this case report of improvement in food intolerance and ASD symptoms after FMT in a male patient constitutes an important insight into a possible involvement of the gut microbiome in the pathogenesis of food intolerances.

## 2 Case report

The patient is a male born in 2017. Family history was negative for allergies or psychiatric disorders.

This study was approved by the Ethics Committee of the First Affiliated Hospital of Guangdong Pharmaceutical University [Approval No. 2923JS (11)]. And the informed consent form for FMT was signed by the patient’s legal guardian (PDF1).

In October 2019, he was diagnosed with “Autistic Spectrum Disorder” due to symptoms including poor language communication skills, irritability, social withdrawal, loose stools, multiple scattered eczema lesions and sleep disturbances. In December 2019, a 90-item food-specific IgG antibody test identified intolerance to 52 foods (Table 1), with severe reactivity to staples like rice, wheat, and milk. Elimination of these foods improved language expression, eczema, and sleep quality. Reintroduction of intolerant foods consistently provoked rashes and sleep disturbances, confirming provocation test results. Additionally, allergen-specific IgE antibody testing for inhaled and ingested allergens showed no significant abnormalities. The delayed diagnosis of FI in this pediatric patient with ASD and communication impairments was attributable to its less overt clinical manifestations compared to food allergy, compounded by age-related diagnostic challenges. The patient adheres to a structured food diary with rotation of tolerated foods. The patient presents with persistent generalized rashes, frequent diarrheal episodes, and an immunocompromised state, leading to multiple hospitalizations for recurrent pneumonia.

**TABLE 1:** Positive items of 90-item food-specific IgG antibody test.

Food	Year	
	2019	2024
Peanuts	Grade 3	Grade 3
Milk	Grade 3	Grade 2
Soybeans	Grade 3	Grade 3
Eggs	Grade 3	Grade 3
Rice	Grade 3	Grade 2
White soft cheese	Grade 3	Grade 1
Wheat	Grade 3	Grade 1
Almond	Grade 2	Grade 3
Yogurt	Grade 2	Grade 0
Sunflower seeds	Grade 2	Grade 2
Mustard	Grade 2	Grade 2
Pumpkin	Grade 2	Grade 1
Barley	Grade 2	Grade 1
Millet	Grade 2	Grade 1
Broccoli	Grade 2	Grade 1
Garlic	Grade 2	Grade 1
Black walnuts	Grade 2	Grade 1
Goat’s Milk	Grade 2	Grade 0
Potatoes	Grade 2	Grade 0
Buckwheat	Grade 2	Grade 0
Tomatoes	Grade 2	Grade 0
Cashew	Grade 2	Grade 3
Mixed Peas	Grade 1	Grade 1
Onions	Grade 1	Grade 1
Cheddar cheese	Grade 1	Grade 1
Cinnamon	Grade 1	Grade 1
Eggplants	Grade 1	Grade 0
Green peppers	Grade 1	Grade 0
Parsley	Grade 1	Grade 0
Cabbage	Grade 1	Grade 0
Carrots	Grade 1	Grade 0
Pineapples	Grade 1	Grade 0
Green beans	Grade 1	Grade 0
Cantaloupe	Grade 1	Grade 0
Rye	Grade 1	Grade 0
Butter	Grade 1	Grade 0
Honey	Grade1	Grade 0
Crab	Grade 1	Grade 0
Shrimp	Grade 1	Grade 0
Cod	Grade 1	Grade 0
Clams	Grade 1	Grade 0
Sardines	Grade 1	Grade 0
Lobster	Grade 1	Grade 0
Oysters	Grade 1	Grade 0
Scallions	Grade 1	Grade 0
Lettuce	Grade 1	Grade 0
Cucumbers	Grade 1	Grade 0
Watermelon	Grade1	Grade 0
Bok choy	Grade 1	Grade 0
Pomelos	Grade 1	Grade 0
Bananas	Grade 1	Grade 0
Oranges	Grade 1	Grade 0
Chili pepper	Grade 0	Grade 1
Corn	Grade 0	Grade 1
Mangetout	Grade 0	Grade 1
Mushroom	Grade 0	Grade 1

In July 2024, the child received oral fecal microbiota capsule treatment.

Dosage and administration: three capsules per dose, three times daily, administered with warm water 30 min prior to meals. Each treatment course consists of 120 capsules (4.2 × 10^13^ CFU/course) (PDF2–Daily Microbial Suspension Logbook), with the therapy to be continued consecutively for two complete courses. Capsule specification: No. 3 pediatric-sized capsules are to be utilized for encapsulation.

The donor microbiota was sourced from a rigorously screened healthy adult who had no comorbidities or disorders known to be associated with changes in gut microbiota, were chosen as donors. Donor stools were screened for enteric pathogens including parasites (Entamoeba histolytica, Giardia) and bacteria (Salmonella, Shigella, Escherichia coli, Campylobacter, Yersinia, and C. difficile). The donors were accepted only if HAV IgM, HBsAg, anti-HCV antibodies, anti-human immunodeficiency virus antibodies, IgM antibodies against cytomegalovirus and tests for syphilis were negative. The stool sample was not accepted if donors had taken antibiotics or probiotics in previous 3 months. FMT possesses repeated microfiltration, centrifugation, washing, discarding resuspension and capsules preparation based on the automatic microfiltration machine (GenFMTer, Nanjing, China) in a biosafety level-3 laboratory (18, 19) and prepared by the Microecology Center of the First Affiliated Hospital of Guangdong Pharmaceutical University. Viable bacterial counts in all capsule preparations were validated to meet international standards (18, 20) prior to lyophilization. Furthermore, proactive donor fecal sample screening was implemented in response to real-time adverse event monitoring, with no FMT-related adverse events reported to date.

**FIGURE 1:**
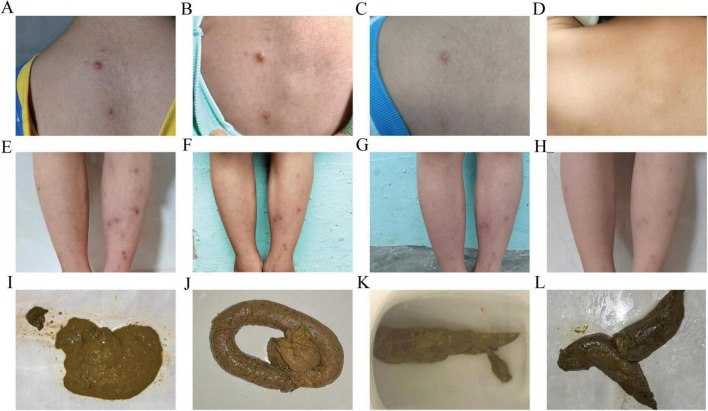
Changes in maculopapular rash and fecal characteristics after taking fecal microbiota capsules. **(A–D)** Maculopapular rash on the trapezius region: **(A)** on 2024-07-22; **(B)** on 2024-08-07; **(C)** on 2024-08-22; **(D)** on 2025-03-22. **(E–H)** Maculopapular rash on both lower legs: **(E)** on 2024-07-22; **(F)** on 2024-08-22; **(G)** on 2024-09-22; **(H)** on 2025-03-22. **(I–L)** Fecal characteristics: **(I)** on 2024-07-21; **(J)** on 2024-08-22; **(K)** on 2024-10-21; **(L)** on 2025-3-22.

September 2024: After two courses of FMT capsules, the child’s symptoms significantly improved. The child tolerated previously intolerant foods (e.g., rice, wheat, soy, peanuts, and milk). No new rashes appeared, the existing rashes resolved (Figure 1), stool consistency normalized (Figure 1), and nutritional status improved with a gradual in-crease in body mass index (BMI) (Table 2).

**TABLE 2:** Changes in body mass index (BMI) after taking in the fecal microbiota capsules.

Time	Height	Weight	BMI
22-07-2024	115	17	12.9
07-08-2024	115	17.7	13.4
22-08-2024	115	18.3	13.8
22-09-2024	116	18.75	13.9
22-10-2024	116	19	14.1
22-11-2024	116	20	14.9
22-12-2024	117	20.5	15
22-01-2025	118	21	15.1
22-02-2025	120	22	15.3
22-03-2025	120	22.2	15.4

During the therapeutic course, the patient exhibited good tolerability and maintained high adherence to the prescribed pharmacological regimen. The legal guardian reported significant improvement in the patient’s rash condition post-capsule administration, with no pruritus or other adverse symptoms, demonstrating good acceptance. No adverse reactions were observed during the treatment.

December 2024: A repeat 90-item food-specific IgG antibody test showed significant improvement after two courses of fecal microbiota transplantation (Table 1). Post-FMT reassessment of food-specific IgG antibodies demonstrated a marked reduction in both the number and magnitude of food intolerances (Table 3).

**TABLE 3:** The alterations in the 90-item food-specific IgG antibody panel were analyzed before and after the administration of fecal microbiota capsules.

Grade	Year	
	2019	2024
Grade 0	38	65
Grade 1	30	16
Grade 2	15	4
Grade 3	7	5

**FIGURE 2:**
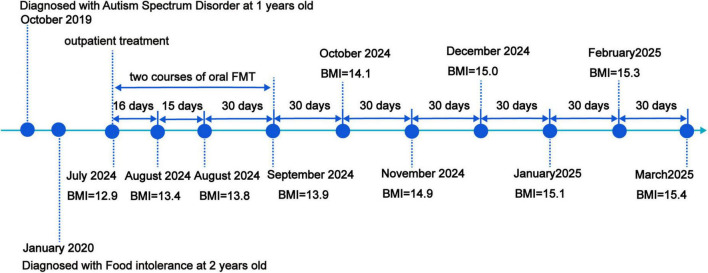
Timeline of clinical findings and nutritional intervention.

The chronological summary of the patient’s previous diagnostic and therapeutic interventions is systematically outlined in Figure 2.

## 3 Discussion

This case report demonstrates notable clinical and methodological advancements in managing severe FI through FMT. Notably, it represents the first documented application of FMT for severe FI (52 items) in pediatric ASD, addressing a critical therapeutic gap in this complex patient population. Furthermore, the non-invasive oral capsule administration protocol overcomes procedural limitations typically encountered in ASD patients, enhancing clinical feasibility while maintaining therapeutic efficacy. Importantly, the intervention achieved multidimensional improvements encompassing cutaneous (rash resolution), gastrointestinal (stool normalization), immunological (restored food tolerance), and nutritional (BMI elevation) domains, suggesting systemic biological effects beyond symptomatic relief. Additionally, the standardized dosing regimen (nine capsules/day × 120 capsules/course × 2 courses) establishes a replicable framework for future trials. This correlation between microbiota modulation and regained oral tolerance aligns mechanistically with emerging evidence on gut microbiome-mediated antigen processing, thereby strengthening the biological plausibility of FMT as a disease-modifying therapy for FI.

Food intolerance can be caused by a variety of mechanisms, including enzyme deficiencies (e.g., lactase deficiency), pharmacological effects, irritant reactions, and toxicological responses. The gut microbiota plays a pivotal role in the pathogenesis of food intolerance.

The gut microbiota is involved in the pathogenesis of food intolerance caused by lactase deficiency. Lactase deficiency leads to undigested lactose interacting with the intestinal microbiota then produce short-chain fatty acids (SCFA) (acetate, propionate) and gases (hydrogen, carbon dioxide). When the amount of lactose exceeds the fermentation capacity of the colonic microbiota, or when the absorption capacity for SCFA in the colon is overwhelmed, diarrhea occurs. Brandao Gois et al. (21) found that, higher *Bifidobacterium* abundance in lactose intolerance individuals (P Wilcox = 4.56 × 10^–9^).

The gut microbiota participates in the pathogenesis of FI associated with Non-Celiac Gluten Sensitivity (NCGS): M. Daulatzai et al. (22) found that NCGS patients exhibit gut microbiota dysbiosis, characterized by reduced beneficial bacteria (e.g., *Bifidobacterium*) and increased pro-inflammatory bacteria (e.g., *Enterobacteriaceae*, *Escherichia coli*, and *Firmicutes*). This dysbiosis may contribute to bloating through enhanced fermentation. Molecular studies highlight increased Claudin-4, a tight junction protein regulating paracellular permeability, supporting the “leaky gut” hypothesis. Hansen et al. (23) demonstrated that an 8 weeks low-gluten diet in 60 Danish adults reduced fecal *Bifidobacterium*, *Dorea*, *Blautia*, *Lachnospiraceae*, and butyrate-producing bacteria (*Anaerostipes hadrus*, *Eubacterium hallii*), alongside decreased postprandial hydrogen breath and bloating.

The gut microbiota is involved in the pathogenesis of FI induced by FODMAPs. FODMAPs, short-chain carbohydrates including lactose, fructose, sugar alcohols, fructans, and galacto-oligosaccharides (GOS), are naturally present in fruits, vegetables, grains, and dairy products. Their malabsorption results from deficiencies in brush border enzymes (e.g., lactase, sucrase) in the small intestine, leading to osmotic water retention and diarrhea. Dysregulated gut microbiota further exacerbates this condition, as pathogenic bacteria ferment undigested FODMAPs in the colon, producing excessive hydrogen, methane, and acidic by-products. This accumulation of gas, liquid, and acids stimulates the intestinal wall, causing FI symptoms such as bloating, abdominal pain, and diarrhea. The gut microbiota is implicated in the pathogenesis of histamine intolerance.

Histamine intolerance arises from impaired histamine degradation due to reduced activity or levels of histamine-metabolizing enzymes. Consumption of histamine-rich foods, or DAO-inhibiting medications elevates exogenous histamine. In these patients, gut dysbiosis is characterized by increased *Proteus* bacteria, damaging epithelial cells and DAO production. Such dysregulation exacerbates endogenous histamine levels, worsening FI symptom (24). Histamine-intolerant individuals also exhibit reduced beneficial bacteria (e.g., *Prevotellaceae*, *Ruminococcus*, *Faecalibacterium prausnitzii*) and increased histamine-secreting bacteria (e.g., *Staphylococcus*, *Proteus*, *Clostridium perfringens*, *Enterococcus faecium*) (25).

The association between gut microbiota dysbiosis and FI is increasingly recognized, highlighting the potential of microbiota-targeted therapies in managing FI. Probiotics and prebiotics have been explored as treatments since 2001 (26), with recent studies supporting probiotic supplementation for lactose intolerance (27). Animal studies further validate their efficacy. Ardizzone et al. (28) demonstrated that a novel therapeutic formulation (NTN) containing *Lactobacillus acidophilus* and *Lactobacillus reuteri* restored intestinal barrier integrity and permeability in mice with diet-induced FI, alleviating related symptoms. Ferrari et al. (29) highlighted probiotics’ role in modulating gut microbiota, reducing ER stress, mitigating inflammation, and enhancing barrier function, collectively improving FI. Additionally, Besseling-van der Vaart et al. (30) showed that the multi-strain probiotic Ecologic^®^ Tolerance (Syngut™) enhanced β-galactosidase activity, strengthened epithelial barriers, and improved resistance to digestive enzymes and bile salts *in vitro*.

These findings collectively underscore the therapeutic potential of probiotics in addressing gut microbiota dysbiosis and alleviating FI symptoms, providing a foundation for further clinical investigations.

After two consecutive treatment of oral fecal microbiota capsule, retesting of 90-food-specific IgG antibodies revealed decreased intolerance levels to most dietary antigens. Paradoxically, cashews and almonds progressed from moderate to severe intolerance, while corn, mushrooms, and capsaicin transitioned from tolerable to mild intolerance. We hereby propose the following discussion points.

Cashew and almond intolerance progressed from moderate to severe. These nuts are rich in lectins, which are resistant to high temperatures and enzymatic digestion in both rodents and humans (31). Emerging evidence highlights that children with ASD frequently exhibit depletion of butyrate-producing commensals, particularly *Faecalibacterium prausnitzii* (32). Post-FMT fluctuations in SCFA concentrations, notably butyrate, may activate GPR41/GPR43 receptors to modulate host energy metabolism and anti-inflammatory responses (33). However, altered intestinal transit time secondary to SCFA shifts could paradoxically prolong luminal exposure to undegraded dietary lectins. Lectins can translocate across the intestinal barrier into the bloodstream, where they deposit on blood and lymphatic vessel walls, stimulating the immune system (34), ultimately leading to elevated IgG levels. Lectins, through binding to glycans on the intestinal mucosa, may disrupt mucin polysaccharide architecture, impairing bacterial adhesion and proliferation (35), while concurrently inhibiting brush-border enzyme activity (e.g., disaccharidases), thereby exacerbating maldigestion and nutrient malabsorption (36, 37). Furthermore, lectin-mediated agglutination of beneficial symbionts may reduce their ecological fitness (38, 39), creating niches for opportunistic pathogens such as *Escherichia coli* and *Lactobacillus* spp. to proliferate. This dysbiosis disrupts microbial equilibrium, thereby contributing to fluctuations in IgG levels (40).

What’s more, New-onset mild intolerance to mushroom, corn, and capsaicin was observed. Notably, chitin—a fungal polysaccharide abundant in mushrooms—alters microbial community structure (41), and incomplete engraftment of donor-derived chitinolytic taxa could manifest as transient reactivity to mushroom components. The metabolism of resistant starch (e.g., maize-derived) relies on *Clostridium butyricum*-encoded amylases (42), whereas capsaicin a TRPV1 receptor agonist, demonstrates tolerability closely linked to gut microbiota composition.(43). FMT-induced dysbiosis may disrupt these specialized metabolic pathways, potentially explaining transient post-FMT intolerances.

Current understanding of post-FMT microbiota engraftment remains incomplete. Analogous to organ transplantation, FMT faces inherent challenges of “microbiota rejection,” wherein host immune and ecological factors limit donor strain persistence (44). Chen et al. demonstrated that donor strain engraftment rates rarely exceed 65%, with most clinical cohorts achieving < 30% colonization (45). While microbial network topology may attain dynamic equilibrium within weeks to 3 months post-FMT, functional stabilization (e.g., metabolic cross-feeding networks) likely requires extended timelines (46), paralleling gradual host physiological recovery. Delayed colonization of keystone taxa (e.g., *F. prausnitzii*) may thus underpin *de novo* food reactivity during this transitional phase.

Above all, these findings indicate that dysbiosis of the gut microbiota plays a significant role in the development of various types of FI. The mechanisms of FI in this case suggest a multifactorial origin of FI. FMT may be an effective intervention to restore microbial balance, reduce fermentation, repair the intestinal barrier, and reduce the levels of pro-inflammatory markers such as IFN-γ and histamine. This treatment likely contributed to the significant improvement in the child’s symptoms, including the resolution of rashes, normalization of stool consistency, and improvement in BMI. The successful clinical response supports the role of microbiota modulation in the treatment of complex FIs.

Fecal microbiota transplantation is a promising therapy for chronic diseases associated with gut microbiota alterations (47). Comparative analysis of FMT across distinct disease entities is necessary. Key mechanistic insights are summarized below: The therapeutic mechanism of FMT in IBS emphasizes gut dysbiosis-driven visceral hypersensitivity, characterized by overproliferation of Gram-negative bacteria such as *Proteus mirabilis* and depletion of probiotics including *Lactobacillus rhamnosus GG*. Dysregulated microbial metabolites, such as LPS, suppress resolvin D1 synthesis in colonic tuft cells via TLR4/MyD88 signaling, perpetuating inflammatory cascades and nociception (48).

In the management of FI,FMT primarily enhances intestinal barrier function and energy metabolism through probiotic engraftment to cure FI. For instance, lactose intolerance improvement correlates with increased *Bifidobacterium* abundance, whose β-galactosidase activity facilitates lactose digestion without generating gas byproducts (e.g., hydrogen, carbon dioxide, methane) that drive bloating.

In the management of ASD,FMT reshapes gut microbiota by enriching beneficial taxa (*Bacteroides fragilis, Lactobacillus reuteri*) while suppressing pathobionts (*Clostridiales, Eubacterium coprostanoligenes*). This modulates neuroactive metabolites (4-EPS, SCFAs) and neurotransmitters (serotonin, dopamine), restoring gut barrier integrity and suppressing inflammation. These effects synergistically ameliorate ASD core symptoms and gastrointestinal comorbidities via vagal nerve and hypothalamic-pituitary-adrenal (HPA) axis cross-talk.

Emerging evidence highlights that increased abundance of *Bifidobacterium* (49–51) in the gut microbiota is associated with clinical improvement in FI, IBS, and ASD. Notably, reduced *Faecalibacterium* (52–54) levels have been consistently reported across these three conditions, while ASD-specific dysbiosis is further characterized by overproliferation of *Sutterella* (55). Current research confirms the therapeutic efficacy of *Bacteroides fragilis* strain BF839 in ASD intervention; however, no specific microbial strains have yet been identified for targeted management of IBS or FI.

However, this study has several limitations. First, this study’s sample size was small, and studies with larger sizes and control group are needed for further exploration. Second, 8 months’ observations can’t fully reflect the effects of FMT on devel-oping FI symptoms. Studies with longer follow-up are needed to characterize the long-term efficacy and safety of FMT for pediatric patients. Finally, This case lacked pretreatment assessment of DAO, zonulin, endotoxin, serum histamine, SCFAs, and gut microbiota composition, The absence of zonulin and LPS measurements in this study may limit comprehensive evaluation of intestinal barrier integrity and systemic inflammation. For instance, Fasano et al. (56) demonstrated that zonulin serves as a sensitive biomarker of intestinal permeability, with dynamic changes reflecting FMT-induced mucosal repair. LPS, a key driver of gut hyperpermeability, inhibits tight junction proteins (e.g., occludin, claudin-1) via the TLR4/MyD88 pathway, directly compromising barrier function (45). The lack of LPS quantification precludes definitive conclusions regarding FMT-mediated LPS reduction and tight junction restoration.

Similarly, SCFA levels—critical mediators of microbiota-driven immune and barrier regulation (57) were not assayed. While clinical improvements (e.g., reduced diarrhea, enhanced behavioral scores) may correlate with SCFA restoration (e.g., butyrate, propionate), the absence of direct SCFA data impedes mechanistic validation. Histamine levels were also unmeasured. Histamine intolerance mechanisms include exogenous intake (high-histamine foods), dysbiosis, intestinal hyperpermeability, gastrointestinal bleeding, or mastocytosis. This omission constrains deeper mechanistic exploration (13).

Diamine oxidase levels hold significant reference value in diagnosing HIT, yet their clinical utility necessitates comprehensive multidisciplinary evaluation. Studies demonstrate that HIT patients exhibit markedly lower serum DAO levels compared to healthy controls, and strict dietary intervention correlates with DAO elevation alongside symptom remission, suggesting DAO as a biomarker for monitoring dietary compliance and therapeutic efficacy (58, 59). However, DAO’s diagnostic sensitivity remains constrained: only 50%–71% of HIT patients present DAO < 10 U/mL, while subnormal DAO levels are also observed in asymptomatic populations, resulting in a low positive predictive value for standalone testing (59, 60). Thus, DAO testing should serve as a complementary tool, integrated with clinical symptomatology, dietary provocation trials, and exclusion diagnostics to enhance diagnostic precision (58, 59, 61). These limitations mirror broader technical challenges in FMT research and underscore the necessity of standardized multi-omics platforms for mechanistic elucidation.

Due to the resource constraints and the patient’s outpatient clinical follow-up protocol, which precluded comprehensive analysis of fecal gut microbiota composition. The absence of microbiota profiling hinders mechanistic exploration of microbial metabolites in symptom amelioration. However, the single-case design inherently limits the statistical power required for microbiota-symptom correlation analyses. Future multicenter trials with serial metagenomic and metabolomic profiling will elucidate microbial drivers of therapeutic responses.

## Data Availability

The original contributions presented in this study are included in this article/Supplementary material, further inquiries can be directed to the corresponding authors.
