# Supplementary material for: Therapeutic efficacy of fecal microbiota transplantation in severe food intolerance: a case report
Source: Front Nutr. 2025 May 19;12:1594022. doi: 10.3389/fnut.2025.1594022 (PMC12127135; doi:10.3389/fnut.2025.1594022)
Supplement: Supplementary file 1 [file Supplementary_file_1.pdf]

CARE case report guidelines

CARE Checklist of information to include when writing a case report

(cc) BY-NC-ND

Topic	Item	Checklist item description	Reported on Line
Title	1	The diagnosis or intervention of primary focus followed by the words "case report"	64-70
Key Words	2	2 to 5 key words that identify diagnoses or interventions in this case report, including "case report"	99-101
Abstract (no references)	3a	Introduction: What is unique about this case and what does it add to the scientific literature?	105-147
	3b	Main symptoms and/or important clinical findings	87-90
	3c	The main diagnoses, therapeutic interventions, and outcomes	90-94
	3d	Conclusion—What is the main "take-away" lesson(s) from this case?	94-96
Introduction	4	One or two paragraphs summarizing why this case is unique (may include references)	141-146
Patient Information	5a	De-identified patient specific information.	152-153
	5b	Primary concerns and symptoms of the patient.	158-167
	5c	Medical, family, and psycho-social history including relevant genetic information	152-153
	5d	Relevant past interventions with outcomes	164-169
Clinical Findings	6	Describe significant physical examination (PE) and important clinical findings.	174-177
Timeline	7	Historical and current information from this episode of care organized as a timeline	473-496
Diagnostic Assessment	8a	Diagnostic testing (such as PE, laboratory testing, imaging, surveys).	158-163, 168-169
	8b	Diagnostic challenges (such as access to testing, financial, or cultural)	169-173
	8c	Diagnosis (including other diagnoses considered)	158-169
	8d	Prognosis (such as staging in oncology) where applicable	
Therapeutic Intervention	9a	Types of therapeutic intervention (such as pharmacologic, surgical, preventive, self-care)	178-179
	9b	Administration of therapeutic intervention (such as dosage, strength, duration)	180-186
	9c	Changes in therapeutic intervention (with rationale)	209-214
Follow-up and Outcomes	10a	Clinician and patient-assessed outcomes (if available)	
	10b	Important follow-up diagnostic and other test results	222-225
	10c	Intervention adherence and tolerability (How was this assessed?)	215-221
	10d	Adverse and unanticipated events	220-221
Discussion	11a	A scientific discussion of the strengths AND limitations associated with this case report	230-236, 319-331, 639-695
	11b	Discussion of the relevant medical literature with references.	337-354, 376-458, 602-638
	11c	The scientific rationale for any conclusions (including assessment of possible causes)	463-472, 517-590
	11d	The primary "take-away" lessons of this case report (without references) in a one paragraph conclusion	591-601
Patient Perspective	12	The patient should share their perspective in one to two paragraphs on the treatment(s) they received	
Informed Consent	13	Did the patient give informed consent? Please provide if requested	Yes ☑ No ☐
